# Liquid Nitrogen Cryotherapy in the Management of Hemangioma of the Tongue

**DOI:** 10.7759/cureus.24683

**Published:** 2022-05-03

**Authors:** Sneha Krishnan, Muthusekhar M.R., Hemavathy Muralidoss, Santhosh P Kumar, Murugesan Krishnan

**Affiliations:** 1 Department of Oral and Maxillofacial Surgery, Saveetha Dental College and Hospital, Chennai, IND

**Keywords:** hemangioma, liquid nitrogen spray, vascular lesions, tongue, cryotherapy

## Abstract

Recurrent bleeding due to mild trauma, tongue biting, poor oral hygiene, and expansion of the lesions causing difficulty in chewing, swallowing, or speaking is common sequelae of vascular lesions of the tongue. Cryosurgery is a technique for destroying lesions by the rapid freezing method. The necrotic tissue that results is allowed to slough naturally after the lesion is frozen. Cryosurgery has been utilized in the medical and dentistry field to treat many lesions as it is successful and easy to perform. It has a number of advantages, including ease of use, minimal infection rate, and no bleeding intraoperatively. This report presents a case of a 15-year-old female patient with a hemangiomatous lesion on the tongue that was successfully treated by cryosurgery using a liquid nitrogen spray. The patient demonstrated complete resolution of the lesion with good wound healing during the one-year follow-up period.

## Introduction

Cryosurgery is a technique for destroying lesions by rapidly freezing them in situ. The necrotic tissue that results is allowed to slough naturally after the lesion is frozen [1]. The ensuing lesion that is subjected to cryotherapy is defined by a neatly delineated necrosis that corresponds to the volume of previously frozen tissue [2]. Cryotherapy was initially used for the treatment of cancers of the lip and mouth and is currently used for the treatment of both benign and malignant skin growths [3]. The essential features of cryosurgery are rapid freezing, slow thawing, and repeating the freeze-thaw cycle [4]. Cryosurgery treatment is effectively utilized in dermatology, proctology, obstetrics, neurology, ophthalmology, general surgery, and head and neck surgery [5].

Cryotherapy is considered the gold standard for treating many lesions in the oral cavity, such as trigeminal neuralgia, mucocele, leukoplakia, pyogenic granuloma, hemangioma, actinic cheilitis, human papillomavirus lesions, fibromas, and lichen planus. It is also considered a supplemental treatment in cases of osseous lesions with high recurrence rates, such as ossifying fibroma, keratocystic odontogenic tumor, central giant cell lesions, myxoma, and ameloblastoma [2]. Tissue reaction varies depending on the severity of the injury with moderate cryogenic injury resulting in an inflammatory response and severe cryogenic injury resulting in tissue destruction. The ultimate goal of cryosurgical treatment is to destroy neoplastic tissues with high levels of precision and spare normal tissue for functional and cosmetic reasons [6]. This report presents a case of a 15-year-old female patient with a hemangiomatous lesion on the tongue that was successfully treated by cryosurgery using a liquid nitrogen spray.

## Case presentation

**Figure 1:**
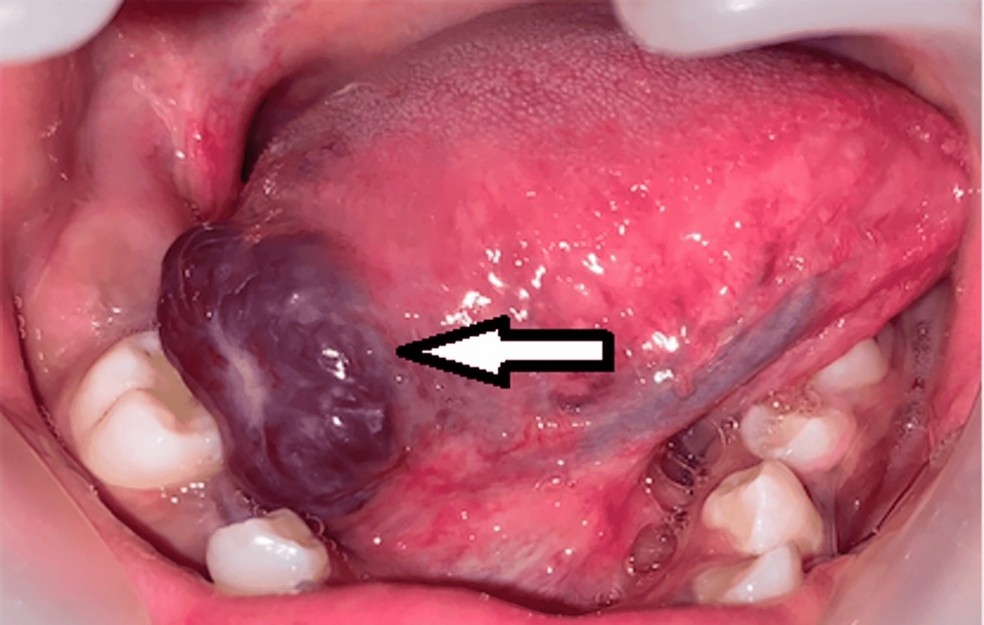
Intraoral view showing lesion on the right lateral border of the tongue (arrow)

A 15-year-old female patient reported to the Department of Oral and Maxillofacial Surgery with a chief complaint of an asymptomatic blue-purplish lesion on the right lateral border of the tongue. History revealed that the onset of the lesion was five months ago and progressed slowly to the present size. There was no family history of malignancy and no episodes of recurrent bleeding from the lesion. Extraoral examination revealed no gross facial asymmetry, and the cervical lymph nodes were not palpable. Intraoral examination revealed an asymptomatic, purplish, sessile, nodular, firm-to-touch lesion measuring about 2.5 cm in its largest diameter on the right lateral border of the tongue (Figure 1). After determining the symptoms of revascularization using finger pressure and obtaining no pulsations from the lesion, the lesion was clinically diagnosed as hemangioma.

**Figure 2:**
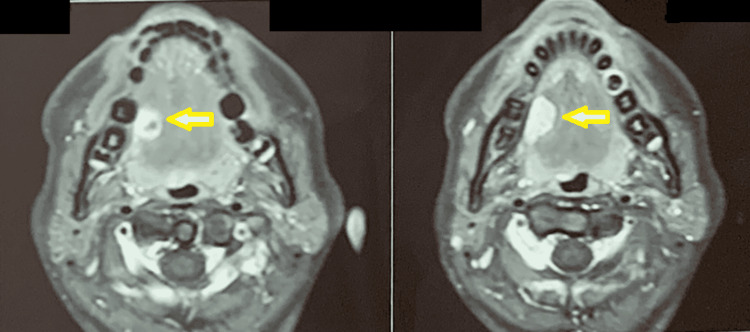
MRI of the neck impression revealing a mildly enhancing, fairly defined, diffusion-restricting, T2/STIR hyperintense lesion with internal hypointensity in the right lateral aspect of the tongue (arrow)

**Figure 3:**
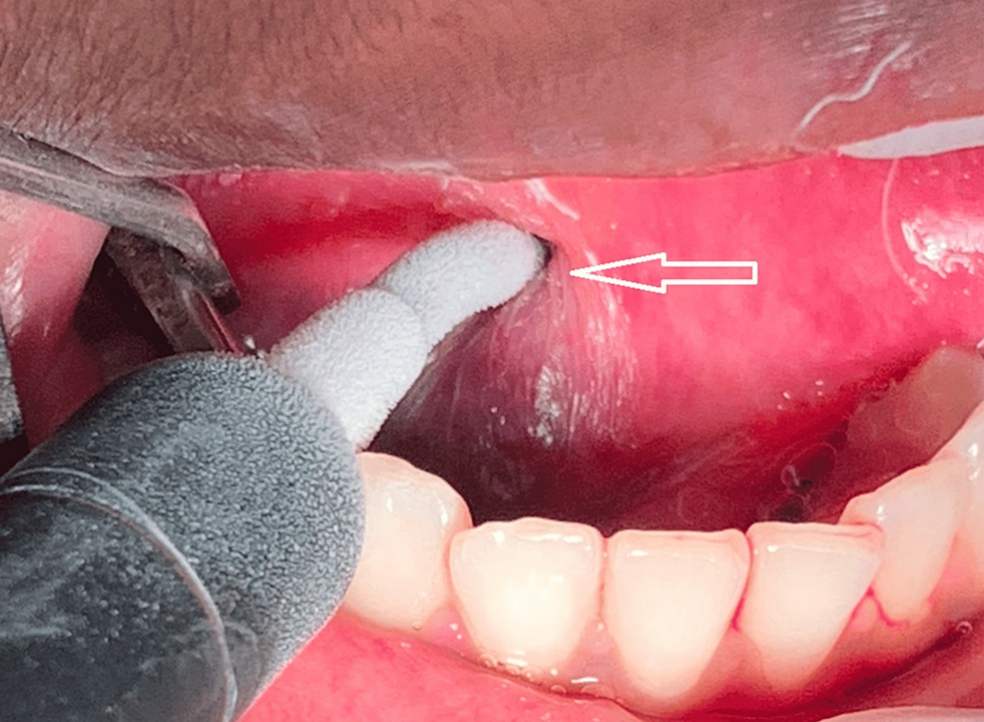
Application of liquid nitrogen through a large contact tip to the lesion (arrow)

Investigations such as contrast-enhanced computed tomography scan, magnetic resonance imaging (MRI) of the neck with contrast, and digital subtraction angiography were done, which revealed the lesion to be a low-flow lesion. MRI of the neck revealed a mildly enhancing, fairly defined, diffusion-restricting, T2/STIR hyperintense lesion with internal hypointensity (calcification) in the right anterior lateral aspect of the tongue involving the intrinsic and extrinsic muscles (Figure 2). Cryosurgery of the lesion was chosen as the treatment modality because of the significant risk of bleeding with other surgical options. Incisional biopsy is contraindicated in hemangiomatous lesions as minor manipulation of the lesion might induce substantial hemorrhage and bleeding. The patient's hemodynamics was stable, and the cryosurgery procedure was conducted in an outpatient setting under a local anesthetic. The surgical site was sterilized with 10% betadine solution, followed by anesthesia of the apex of the tongue and afflicted area by an anesthetic block of the lesion site with lignocaine with epinephrine (1:200,000). The tongue apex was sutured with 3-0 silk and moved to the left, and Vaseline-soaked gauze was used to protect the surrounding tissues (tongue, cheek mucosa, palate, and teeth). The cryosurgery was performed with liquid nitrogen spray in four sessions of six cycles of 30 seconds each, with an interval of two minutes between cycles (Figure 3). Thawing proceeded spontaneously for 30-60 seconds thereafter. Six consecutive freeze-thaw cycles were used. High-speed suction was used during treatment to control visual obstruction from vapor fog.

**Figure 4:**
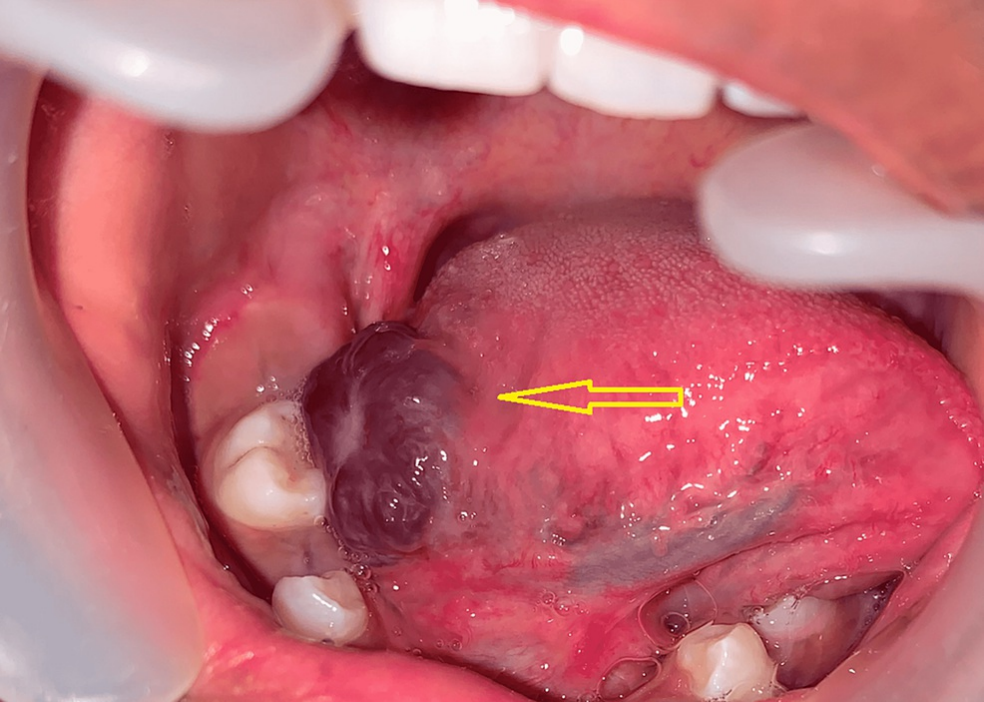
Lesion (arrow) after the first session of cryotherapy (postoperative first week)

**Figure 5:**
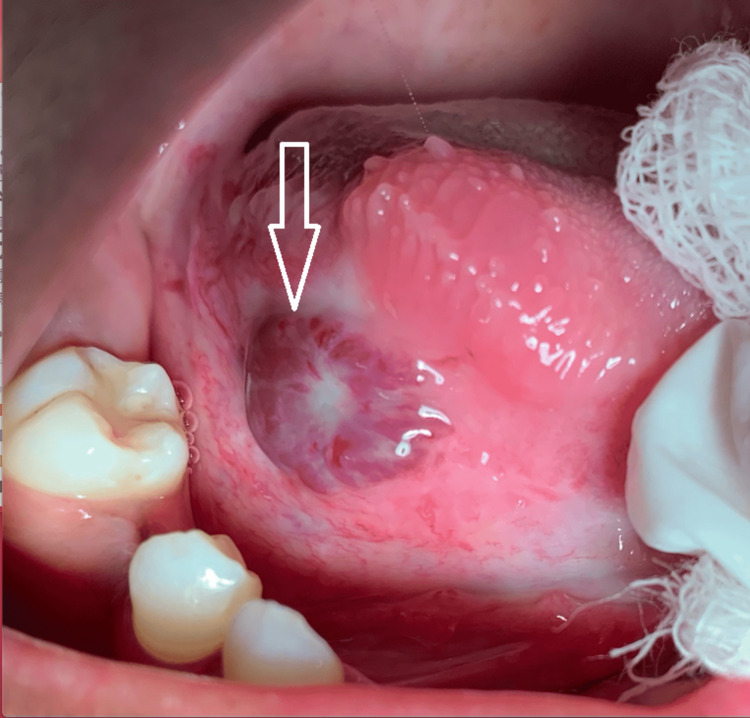
Lesion (arrow) after the second session of cryotherapy (postoperative third week)

**Figure 6:**
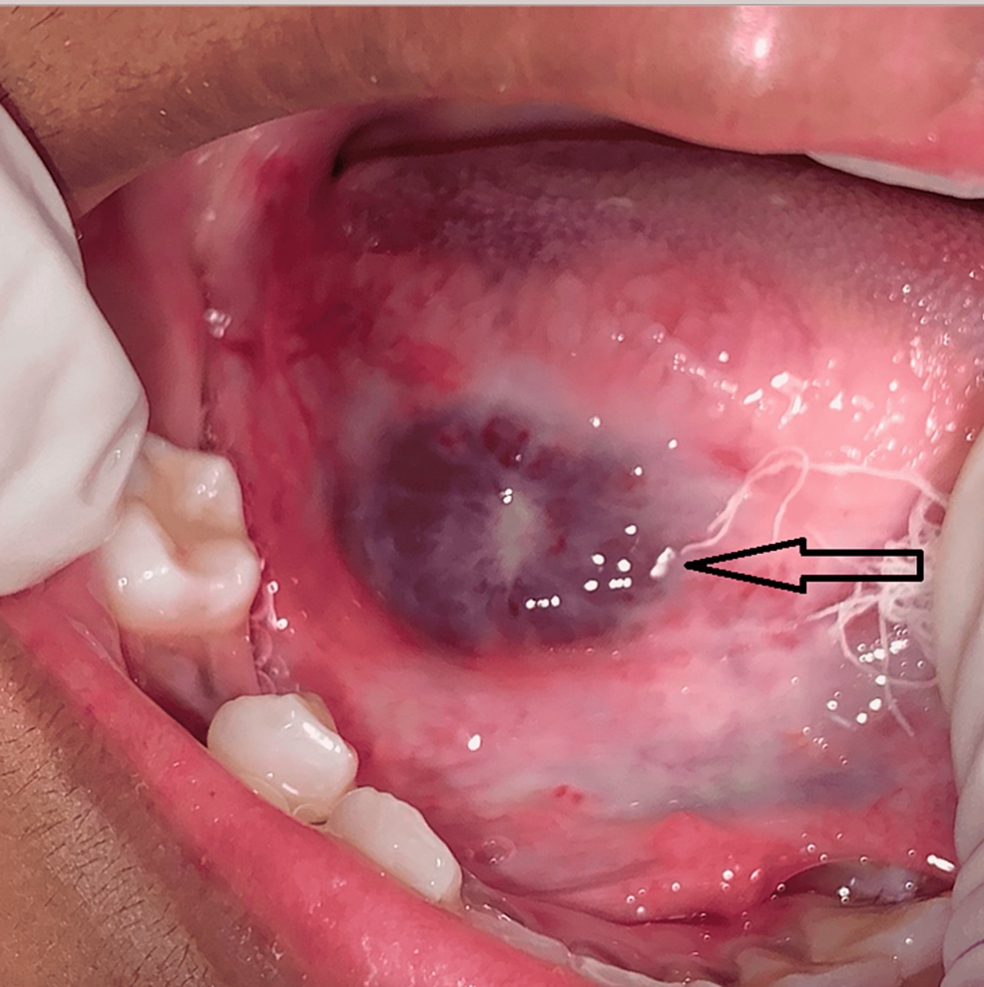
Lesion (arrow) after the third session of cryotherapy (postoperative sixth week)

**Figure 7:**
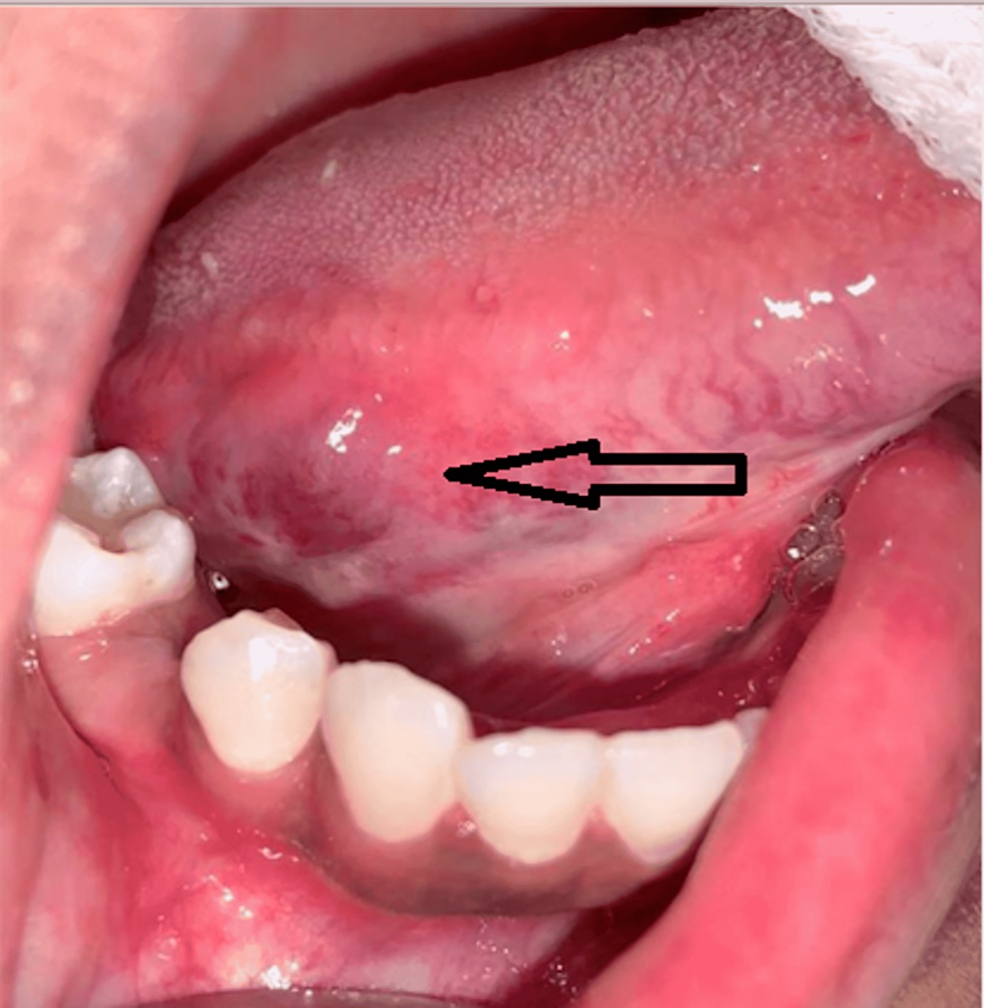
Lesion (arrow) after the fourth session of cryotherapy (postoperative ninth week)

The lesion appeared whitish in color immediately after the procedure, indicating the freezing process, and edema and erythema formation were visible in the treated area a few minutes later after thawing. The dentition was spared from contact with the liquid nitrogen, and it was ensured that the spray did not cause any liquid to collect and drain into the pharynx. Four sessions of liquid nitrogen cryotherapy were delivered at periodic intervals. The patient was periodically reviewed postoperatively at the first, third, sixth, and ninth weeks following each session of cryotherapy (Figures 4-7).

**Figure 8:**
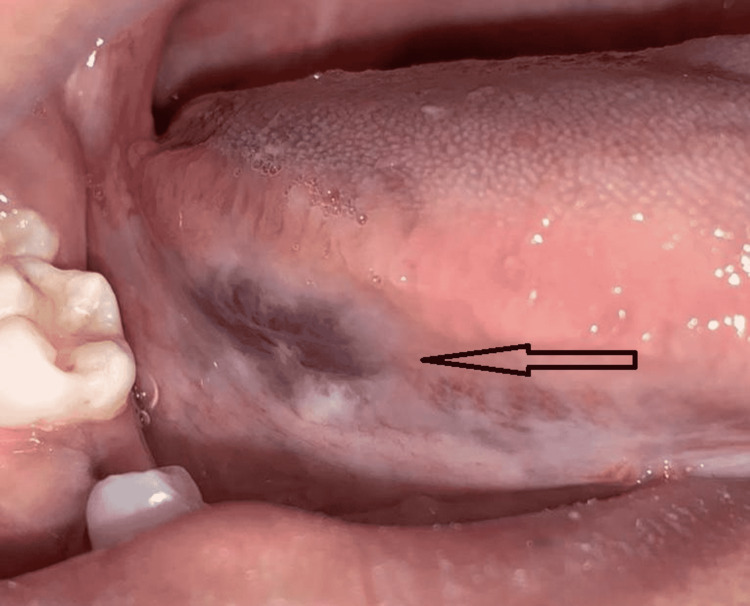
Regression of the lesion with good wound healing present during the one-year postoperative follow-up period (arrow)

The patient experienced slight pain in the first few days after surgery, which was treated with oral analgesics for three days. During the healing process, there were no signs of infection. The patient was clinically healthy during the one-year postoperative follow-up period with no sensory abnormalities in the tongue, aberrant speech, or difficulty swallowing and no symptoms of lesion recurrence (Figure 8). However, a small scar retraction was present at the lesion location.

## Discussion

Hemangiomas commonly occur in early childhood and infancy [7]. It is a true vascular tumor that develops from a neoplastic overgrowth of normal vascular tissue, which later grows by endothelial proliferation. Vascular malformations are caused by aberrant vascular or lymphatic vessel morphogenesis rather than as a result of defective endothelium development [8]. Hemangiomas are benign lesions that spontaneously regress and disappear by the age of seven years, with 2% of these lesions requiring therapy in order to be reduced or removed [9]. Vascular malformations grow slowly and do not involute throughout life, with an increase in reaction to infection, trauma, or hormonal fluctuations. Osseous involvement in hemangiomas is rare; however, osseous involvement is seen in 35% of vascular malformations [10].

Hemangiomas are hamartomatous lesions that are divided histologically into three types based on the size of the proliferating vascular spaces: capillary, cavernous, and mixed [11]. The cavernous type is the most common intraoral hemangioma affecting the buccal mucosa, tongue, and lips [9]. The treatment options are numerous, with varying levels of morbidity. Surgical excision of the hemangiomatous lesions is challenging due to the vascularity and tendency to bleed during the procedure. Sclerosing drugs require multiple injections at 6-8 weeks intervals over a long period of time, and radiation of the areas can cause xerostomia, osteoradionecrosis, and malignant transformation of benign tissues [12].

Cryosurgery has been used successfully in a variety of specialties such as general surgery, ophthalmology, oncology, hepatology, dermatology, urology, and oral and maxillofacial surgery [5-7,11-13]. Histological examination is required to confirm the diagnosis of the lesion; however, biopsy prior to cryotherapy may affect the final outcome for clinically diagnosable lesions such as mucoceles and hemangiomas [3]. Cryotherapy was shown to be a simple and effective treatment modality in the management of a variety of soft tissue lesions affecting the oral cavity, such as hemangioma, leukoplakia, and squamous cell carcinoma [14,15].

To increase tissue damage, cryotherapy's basic technique includes rapid cooling, gradual thawing, and repeated freezing [16]. A closed system using probes and nitrous oxide or an open system using a liquid nitrogen spray or a cotton tip are the two techniques employed in cryosurgery. The majority of tissues freeze at -2.2°C, while tissue death occurs at -20°C. Cryosurgery has the capacity to completely remove a vascular lesion in a single treatment while maintaining hemostasis, good aesthetics, little scar contracture, and minimal patient discomfort. Rapid intracellular crystallization causes cellular dehydration, hazardous electrolyte concentrations, cell membrane rupture, and protein denaturation, which leads to tissue destruction resulting in predictable nonselective tissue damage [17].

Cryotherapy is regarded as a simple, rapid, and easy-to-execute procedure as a result of the prompt appearance of erythema and edema in the cryogenic exposed area. Following cryosurgery, there have been no reports of infection, respiratory issues, or adverse responses to the cryogenic agent. Mild pain was noted in cases, and it was treated primarily with peripherally acting analgesics [15]. Cryosurgery systems employ liquid nitrogen (-196°C) and nitrous oxide (-89°C) as coolants. Liquid nitrogen is the preferred agent for cryotherapy as it is relatively cost-effective and easy to handle and attains a very low temperature (-196°C) [16]. A long-term follow-up of cases revealed that cryotherapy of oral and benign mucosal lesions was well tolerated by patients [18]. Our patient tolerated cryotherapy very well, resulting in good wound healing.

## Conclusions

Cryosurgery is a safe, comfortable, cost-effective procedure and readily accepted by patients. Liquid nitrogen cryotherapy can be utilized as the treatment of choice for vascular tumors such as hemangioma as it has many advantages over conventional surgery. Cryotherapy is currently the most preferred and effective therapeutic option for many benign mucosal lesions.
